# Proteomic characterization of peanut flour fermented by *Rhizopus oryzae*

**DOI:** 10.1016/j.heliyon.2024.e34793

**Published:** 2024-07-21

**Authors:** Christopher P. Mattison, Rebecca A. Dupre, Kristen Clermont, John G. Gibbons, Jae-Hyuk Yu

**Affiliations:** a U.S. Department of Agriculture, Agricultural Research Service, Southern Regional Research Center, FPSQ, New Orleans, LA, 70124, USA; b Oak Ridge Institute for Science and Education, U.S. Department of Energy, Oak Ridge, TN, 37831, USA; c Department of Biology, La Salle University, Philadelphia, PA, 19141, USA; d Department of Food Science, College of Natural Sciences, University of Massachusetts, Amherst, MA, 01003, USA; e Department of Bacteriology, University of Wisconsin-Madison, Madison, WI, 53706, USA

**Keywords:** Food allergy, Peanut, Allergen, *Rhizopus oryzae* (*R oryzae*), Fermentation, Proteomics, Antibody

## Abstract

Fermentation alters the protein content and composition of foods. To characterize fungal catabolism of peanut proteins, defatted peanut flour was fermented by *Rhizopus oryzae* (*R*. *oryzae*) for up to 48 h and evaluated by SDS-PAGE, mass spectrometry, and antibody binding. A clear change in peanut protein migration was observed by SDS-PAGE after 16 h of fermentation. Mass spectrometric analysis indicated changes in allergen peptides and *R. oryzae* proteins. Several low molecular weight allergen fragments produced during fermentation were identified by mass spectrometry. Immunoassays using anti-peanut allergen antibodies demonstrated reduced allergen content as early as 16 h of fermentation. However, ELISA with peanut allergic IgE indicated only slightly reduced allergen binding even after 48 h. These results indicate that while *R. oryzae* fermentation efficiently metabolizes peanut allergens, significant IgE binding remains in lower molecular mass peptides, and therefore *R. oryzae* fermented peanut products would not be safe for peanut allergic individuals.

## Highlights

• *R. oryzae* metabolizes peanut allergens into peptide fragments.
• Peanut allergen reduction varies depending upon the specific allergen.
• IgE-peanut allergen binding is only slightly reduced by *R. oryzae* fermentation.
• *R. oryzae* fermented peanut is not safe for individuals with peanut allergy.

## 1 Introduction

Food allergies are estimated to affect at least 1–3% of people from ‘Westernized’ societies, and represent significant economic, medical, and social burdens [1]. Food allergies are the result of an inappropriate immune reaction in which immunoglobulin E (IgE) binds to a normally harmless food protein [2]. Relatively recent studies have resulted in changes in medical guidance and support for early peanut introduction in infants as a prophylactic measure [3,4]. Further, defatted peanut flour has been approved by the United States Food and Drug Administration (FDA) as a drug for the treatment of peanut allergy [5].

Several factors including environmental changes, lifestyle factors, increased antibiotic use, and consumption of processed and prepackaged foods with relatively little dietary fiber are thought to have contributed to the increased incidence of food allergy in recent years [[6], [7], [8]]. Peanuts are one of eight foods that commonly cause food allergies, and there are three immunodominant peanut allergens; Ara h 1, Ara h 2 (and the highly similar Ara h 6), and Ara h 3 [9]. Processing can also affect the ability of peanuts, other legumes, and tree nuts to bind IgE and elicit an allergic reaction [10]. For example, heating can reduce IgE binding to some peanut allergens and can also alter their capacity to elicit inflammatory mediator release [11,12].

Fermented foods, such as tempeh, are popular in vegetarian and vegan diets. Fermentation can be performed in solution or in a solid-state format depending on the substrate or the desired product. Solid-state fermentation, under limiting water conditions, may be more efficient and have advantages in terms of enzymatic activity or stability associated with the process [13]. Most often, tempeh production involves fermenting cooked soybeans with fungi from the genus *Rhizopus*, and then forming the fermented product into a dense cake that can be eaten raw or used in cooking.

The *Rhizopus* genus of fungi contains at least eight species that are ubiquitous in the environment and include the black bread mold, *Rhizopus stolonifera* [14]. Species including *Rhizopus microsporous* var. *oligosporous* and *R*. *oryzae* are edible and commonly used for in-home food preparation and industrial purposes such as the production of commercial enzymes, organic acids, and many traditional foods including tempeh, porridge, beverages, and some baked dishes [15]. *R. oryzae* and *R. microsporous* are generally recognized as safe (GRAS) by the US FDA. While they have been used for centuries, *Rhizopus* species are more recently being engineered for industrial purposes including the production of organic compounds such as lactic acid, fumaric acid, and ethanol, as well as for the production of enzymes including glucoamylases, cellulases, and tannases [16].

The effects of allergen catabolism during fermentation have been explored in soybeans, and evidence of hydrolyzed proteins and decreased *in vitro* IgE binding of fermented soybeans (relative to non-fermented soybeans) was observed [[17], [18], [19], [20]]. A 2020 review article highlighted the potential of fermentation processes to be utilized for enhancing the sensory qualities of seed protein-based foods, but acknowledged that the effects of fermentation on allergenicity need further investigation [21]. Herein, we used a proteomic approach to evaluate *R. oryzae* catabolism of peanut allergens, and immunoassays to assess anti-peanut allergen antibody binding.

## 2 Materials and methods

### 2.1 Materials

Bovine serum albumin (BSA) and phenylmethylsulfonyl fluoride (PMSF) were purchased from Millipore Sigma (Burlington, MA, USA). “Real Tempeh” *R*. *oryzae* spores were purchased from culturesforhealth.com (www.culturesforhealth.com). Defatted peanut flour was purchased online from Byrd Mill (http://byrdmill.com/). SDS-PAGE 10–20 % tricine gels, 4X NuPAGE LDS sample buffer with reducing agent, and Safe Stain were purchased from Life Technologies (Carlsbad, CA, USA). Anti-Ara h 1 and anti-peanut allergen antibodies were a kind gift from Agrisera (Vännäs, Sweden). Anti-Ara h 2 antibodies were purchased from Indoor Biotech (Charlottesville, VA, USA), and plasma from five peanut-allergic volunteers was obtained from PlasmaLab International (Everett, WA, USA). Biotinylated anti-human IgE was purchased from Southern Biotech (Birmingham, AL, USA), and fluorescently labeled antibodies and streptavidin were purchased from LI-COR (Lincoln, NE, USA).

### 2.2 Peanut flour fermentation

One gram of peanut flour and 0.1 g of *R*. *oryzae* (NRRL 2710) tempeh starter spores were pre-mixed, added to 2 mL of sterile room temperature distilled water, and gently mixed into a paste. The peanut flour and spores were mixed well in a small sterile weigh boat and carefully placed into a polyethylene bag containing 5 mL of water in the bottom to create a humidified chamber. A control (“0 h”) time point was made by removing 0.1 g of the mixture. Small holes were sliced into the top of the bag with a razor blade to allow air flow, and it was placed into a 36 °C incubator. At 4, 8, 16, 24, and 48 h of fermentation, 0.1 g of the mixture was removed and stored at −80 °C. Frozen samples were thawed and resuspended with the addition of 1 mL ice cold sodium borate buffer (0.1 M H_3_BO_3_, 0.025 M Na_2_B_4_O_7_, 0.075 M NaCl, pH 8.6) containing 1 mM PMSF, followed by gentle vortexing to ensure resuspension but not to lyse fungal cells. Samples were incubated for 1 h at 4 °C on a nutator, spun for 15 min at 16,873 relative centrifugal force (RCF) in a microcentrifuge, and the supernatant was collected. Protein content for each sample was quantified by Bradford assay using a BSA standard curve for reference and samples were then frozen at −80 °C.

### 2.3 SDS-PAGE

SDS-PAGE samples containing fermented samples were prepared with 4X NuPAGE LDS (Life Technologies, Carlsbad, CA, USA) sample buffer and 5 μg of protein was added per lane after heating at 65 °C for 15 min in the presence of 5 mM DTT. Gels were electrophoresed using a Novex Mini Cell gel rig (Life Technologies, Carlsbad, CA, USA) and protein migration was followed using prestained Precision Plus molecular weight markers (Bio-Rad, Hercules, CA, USA). Protein migration was visualized using Safe Stain according to the manufacturer's rapid microwave procedure, and images were collected with the 680 nm channel of an Odyssey CLx infrared imaging system (LI-COR, Lincoln, NE, USA). Protein loading was quantified with the captured image to ensure equivalent amounts of protein in each lane based on the 680 nm signal.

### 2.4 Liquid chromatography–mass spectrometry

Soluble borate buffer extracted protein samples were used for the 0, 16, 24, and 48 h time point mass spectrometric analysis, while mass spectrometric analysis of excised and digested gel bands were done for the 0, 32, and 48 h time point samples. All mass spectrometry samples were prepared as described in Clermont et al., 2023 [22], with the alterations described below. Liquid borate-buffer solubilized protein samples from 0-, 16-, 24-, and 48-h time points were reduced with dithiothreitol, alkylated with iodoacetamide, and digested with sequencing-grade modified trypsin (Promega, Madison, WI, USA). Duplicates of soluble protein digests were generated from two separate sample preparations during the study (n = 4 for all time points).

Protein bands excised from SDS-PAGE (0, 32, and 48 h time point samples) were diced into small cubes, and rinsed sequentially in water, 100 mM ammonium bicarbonate, and 50 % acetonitrile. After drying, reduction with dithiothreitol, and alkylation with iodoacetamide, samples were incubated with sequencing-grade modified trypsin (Promega, Madison, WI, USA) for 16 h at 37 °C with gentle agitation. The supernatant and sequential 25 mM ammonium bicarbonate and 5 % formic acid extractions were collected from each sample. The supernatants and extractions for each sample were pooled and dried under vacuum. Samples were re-suspended in 5 % formic acid and analyzed using an Agilent 1200 LC system with an Agilent Chip Cube interface and an Agilent 6520 Q-TOF tandem mass spectrometer (Agilent Technologies, Santa Clara, CA, USA).

Chromatographic peptide separation was accomplished using an Agilent (Santa Clara, CA, USA) chip consisting of a 160 nL enrichment column and a 150 mm analytical column packed with C18 (5 μm beads with 300 Å pores). One microliter aliquots of samples were transferred to the enrichment column via the capillary pump operating at a flow rate of 4 μL/min; the nano pump was run at a flow rate of 600 nL/min. The mobile phase solvents consisted of A: 0.1 % formic acid in H_2_O, and B: 0.1 % formic acid in 90 % CH_3_CN/10 % H_2_O. Peptide elution was achieved using a solvent gradient: 97 % solvent A was gradually decreased to 60 % over 20 min, then decreased to 20 % solvent A over 2 min, and then held for 3 additional minutes.

The MS source was operated at 300 °C with 5 L/min N_2_ flow and a voltage of 175 V. Collision energy varied as a function of mass and charge using a slope of 3.7 V/100 Da and an offset of 2.5 V. An initial MS scan was performed from *m*/*z* 300 to 1600 and up to three multiply charged ions were automatically selected for MS/MS analysis. Following the initial runs, subsequent injections were made excluding ions previously targeted in the prior MS/MS analysis. LC chromatograms and mass spectra were analyzed using MassHunter Qualitative Analysis software (Version B.0301; Agilent Technologies).

Data were processed and searched using Mascot Distiller software (version 2.8.0, Matrix Science, Boston, MA, USA). A custom *Arachis hypogaea-*specific library was used to identify peanut allergens. A genus-specific *Rhizopus* library was generated from the National Center for Biotechnology Information (NCBI) protein database. Searches were performed using peptide and fragment mass tolerances set at 20 and 50 ppm, respectively. Protein scores reported in Table 1, Table 2 provide an indication of protein abundance, but are not quantitative. Mascot protein scores were calculated based upon the probability that observed mass values may match those of a candidate protein randomly, and the reported scores represent the inverse of the probability that the match between the observed mass and candidate peptide were due to chance [23,24].

Carbamidomethylation of cysteine was set as a fixed modification, and oxidation of methionine was included as a variable modification. The digestion enzyme was specified as trypsin or semi-trypsin, and up to two missed cleavages were allowed. For identification of allergens in the soluble protein samples, duplicate sample results were merged into one peak list. This approach was used for both sets of soluble protein digests. Mascot Distiller Quantitation Toolbox was used to perform label-free quantitation using the replicate protocol. Microsoft Excel was used to calculate p-values (two-sample *t*-test) and peptide intensity ratios from the resulting dataset.

### 2.5 Immunoblot

Following SDS-PAGE, proteins were transferred to PVDF membranes, and the membranes were blocked for 1 h at room temperature in phosphate-buffered saline (pH 7.4, PBS) containing 0.1 % Tween-20 (PBST) and 1 % (w/v) bovine serum albumin. Membranes were then incubated with a 1:1000 dilution of primary anti-peanut allergen antibody for 1 h at room temperature. The primary antibody dilution was removed, and the membranes were washed three times for 5 min with 5 mL of PBST, and then incubated for 30 min at room temperature with IRdye-680-labeled secondary antibody from an appropriate host diluted 1:10,000 in PBST. The secondary antibody dilution was decanted, and the membranes were again washed three times with PBST as above. Antibody-binding signal (IRDye) was visualized using an Odyssey CLx instrument (LI-COR, Lincoln, NE, USA).

### 2.6 ELISA

Direct ELISA was used to compare antibody binding to peanut allergens prior to and during fermentation. Plate wells were coated with 5 μg of protein solubilized in 50 μL of sodium carbonate buffer (0.015 M Na_2_CO_3_, 0.035 M NaHCO_3_, pH 9.6) and incubated overnight (16 h) at 4 °C. Coated plate wells were blocked for 1 h at 37 °C with 1 % BSA dissolved in PBST. Excess blocking solution was washed away with three 200 μL washes with PBST. Anti-peanut allergen antibodies (50 μL/well) were added to plate wells at a 1:1000 dilution in PBST, and volunteer anti-peanut allergic samples were used at a 1:5 dilution in PBST for 1 h at 37 °C. Excess primary antibody was washed away with three 200 μL washes with PBST. For anti-peanut allergen antibodies 50 μL of host appropriate IRDye680-labeled secondary antibody (diluted 1:5000 in PBST) was added to plate wells and incubated 30 min at 37 °C. Excess secondary anti-peanut allergen antibody was washed away with PBST (three X 200 μL), and signal was quantified on a LI-COR Odyssey CLX. For human anti-peanut allergic samples, wells were washed three times as above and then incubated 30 min at 37 °C with biotinylated anti-human IgE diluted 1:1000 in PBST. After three washes with PBST, IRdye-680-labeled streptavidin (1:10,000 in PBST) was added to wells for 30 min at 37 °C, and following three final washes signal was quantified on a LI-COR Odyssey CLX.

### 2.7 Statistical analysis

Mass-spectrometry data was evaluated using Microsoft Excel to calculate p-values (p ≤ 0.05, using two-sample *t*-test) and peptide intensity ratios from the resulting dataset. ELISA data were plotted as the average of at least four biological replicates. Error bars were represented as a standard deviation (SD) percentage using the following formula, SD(%) = SD(RFU)/RFU*100, where SD(%) is the standard deviation of the percentage, while SD(RFU) is the standard deviation of the RFU.

## 3 Results

### 3.1 SDS-PAGE profile of fermented peanut flour proteins

**Fig. 1:**
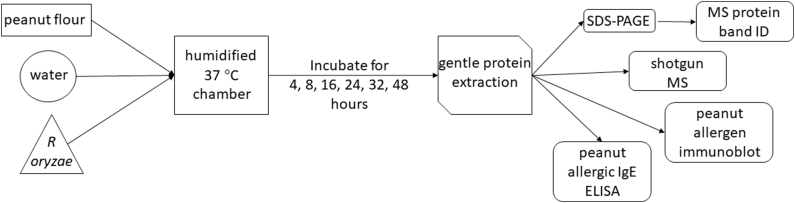
Flow chart for generation and analysis of *R. oryzae* fermented peanut flour.

Peanut flour mixed with *R. oryzae* spores and minimal water was fermented for 48 h at 36 °C in a humidified chamber to evaluate changes in peanut allergen composition (Fig. 1).

**Fig. 2:**
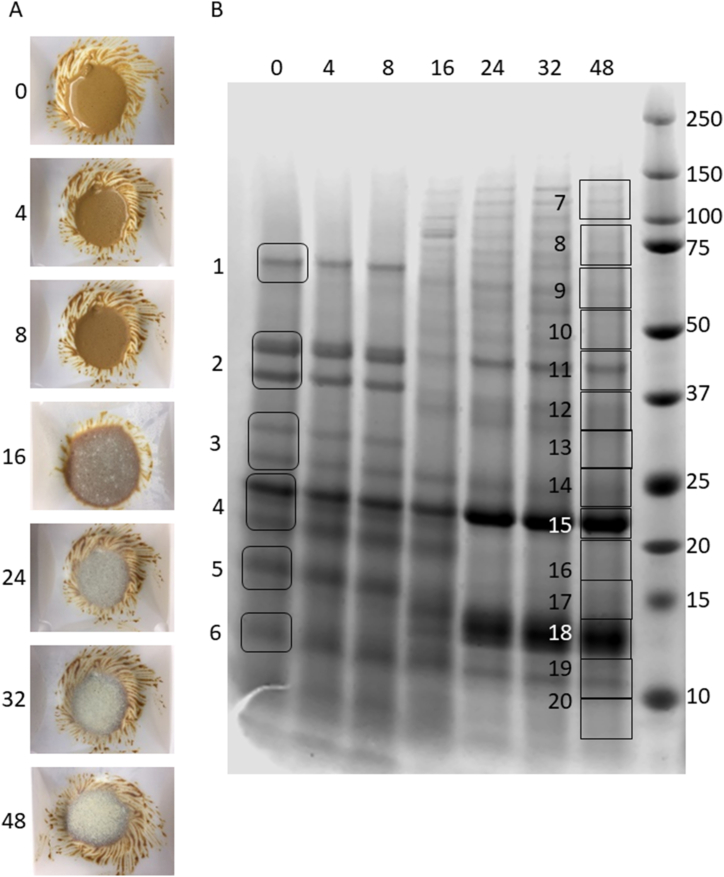
Images of *R. oryzae* fermented peanut flour (A) and Coomassie stained SDS-PAGE protein profiles containing 5 μg of protein per lane (B) following 0, 4, 8, 16, 24, 32, and 48 h of incubation (indicated at top of gel) at 36 °C in a humidified chamber. Numbered boxes indicate excised regions of the gel used for mass spectrometric analysis. Molecular mass markers (kDa) are indicated to the right of the gel.

The mixture resulted in visible fungal growth after 16 h of incubation (Fig. 2A).

SDS-PAGE of soluble protein extracted from the fermentation samples (5 μg per lane) indicated a clear shift in the soluble protein profile between 16 and 24 h (Fig. 2B). For example, the Ara h 1 band migrating at approximately 60 kDa (Fig. 2B, box 1) became less intense and was not discernible after 16 h of fermentation. Similar dynamics were observed in bands corresponding to the acidic (Fig. 2B, box 2) and basic subunits of Ara h 3 (Fig. 2B, box 3 and 4). This portion of the gel showed decreased band intensity as fermentation time elapsed, but at 24 h an approximately 23 kDa band (Fig. 2B, box 15) increased in intensity, presumably due to fungal protein expression. In contrast, the lower molecular weight regions of the gel (<20 kDa) containing Ara h 2 and Ara h 6 (Fig. 2B, boxes 5 and 6) appeared to remain relatively unchanged up to 16 h. However, this region of the gel also displayed a band of approximately 13 kDa (Fig. 2B, box 18) that increased in intensity at 24 h and thereafter.

### 3.2 Mass spectrometric characterization of protein bands

Following band excision and trypsin digestion, proteins from the SDS-page gel bands (Fig. 2B, boxes 1–6) were analyzed using mass spectrometry to confirm protein identities. The peptides identified in the mass spectrometric analyses were searched against libraries containing either peanut or *Rhizopus* protein sequences. The major peanut allergens (Ara h 1, 2, 3, and 6) were identified at their predicted respective masses from the unfermented ‘0 h’ time point (Table 1 and Fig. 2B) [9]. The same gel segments were excised at 32 h of fermentation as indicated by the boxes at 48 h, but they are not indicated on Fig. 2B for clarity. Identification of proteins in 32 and 48 h fermented samples showed Ara h 1 and Ara h 3 peptides in lower molecular weight regions of the gel than initially observed. Table 1 summarizes the key proteins identified in each gel band (Fig. 2B), along with associated Mascot scores and the total percentages of specific proteins identified (% of protein identified). Identified proteins with the top-ranking Mascot scores are presented in Table 1, though many more statistically significant identifications were found in the majority of gel bands (supplementary file 1).

**Table 1:** Mascot scores and percent coverage values of top-ranking peanut allergens identified in SDS-PAGE gel bands by LC-MS/MS analysis of tryptic digests (corresponding gel labels are shown in Fig. 2B). Percent coverage (% cov) indicates the percent of amino acids within the full-length protein that were assigned by high confidence spectral data.

0 h samples	allergen *(accession)*		32 h samples	allergen *(accession)*		48 h samples	allergen *(accession)*	
	score	% cov		score	% cov		score	% cov
			**7**	Ara h 1 *(AAB00861.1)*		**7**	unidentified	
				419	16			
			**8**	Ara h 1 *(AAB00861.1)*		**8**	Ara h 3 *(AAC63045.1)*	
				351	14		172	7
**1**	Ara h 1 *(AAB00861.1)*		**9**	Ara h 1 *(AAB00861.1)*		**9**	Ara h 3 *(AAT39430.1)*	
	1959	31		676	18		28	3
			**10**	Ara h 3 *(AAR02860.1)*		**10**	Ara h 3 *(ABF93402.1)*	
				743	17		294	7
**2**	Ara h 3 *(AAG01363.1)*		**11**	Ara h 3 *(AAU21490.1)*		**11**	Ara h 3 *(AAR02860.1)*	
	2302	49		1592	32		688	17
			**12**	Ara h 3 *(AAR02860.1)*		**12**	Ara h 3 *(AAR02860.1)*	
				875	19		705	18
**3**	Ara h 3 *(ABL14270.1)*		**13**	Ara h 3 *(AAG01363.1)*		**13**	Ara h 3 *(AAG01363.1)*	
	2067	49		1221	25		783	20
			**14**	Ara h 3 *(AAR02860.1)*		**14**	Ara h 3 *(AAR02860.1)*	
				1415	24		749	17
**4**	Ara h 3 *(ABL14270.1)*		**15**	Ara h 3 *(AAU21490.1)*		**15**	Ara h 3 *(AAU21490.1)*	
	1880	23		3412	38		2443	38
			**16**	Ara h 3 *(AAR02860.1)*		**16**	Ara h 3 *(AAG01363.1)*	
				552	19		302	13
**5**	Ara h 6 *(AAL37561.1)*		**17**	Ara h 3 *(AAM46958.1)*		**17**	Ara h 3 *(AAU21491.1)*	
	1427	53		208	15		362	15
			**18**	Ara h 3 *(AAG01363.1)*		**18**	Ara h 3 *(ABL14270.1)*	
				2042	35		987	24
**6**	Ara h 6 *(AAL37561.1)*		**19**	Ara h 6 *(AAL37561.1)*		**19**	Ara h 3 *(AAR02860.1)*	
	507	58		297	31		312	13
			**20**	Ara h 3 *(AAM46958.1)*		**20**	Ara h 3 *(AAM46958.1)*	
				188	6		66	3

### 3.3 Mass spectroscopic analysis of trypsin-digested soluble peanut allergens

**Fig. 3:**
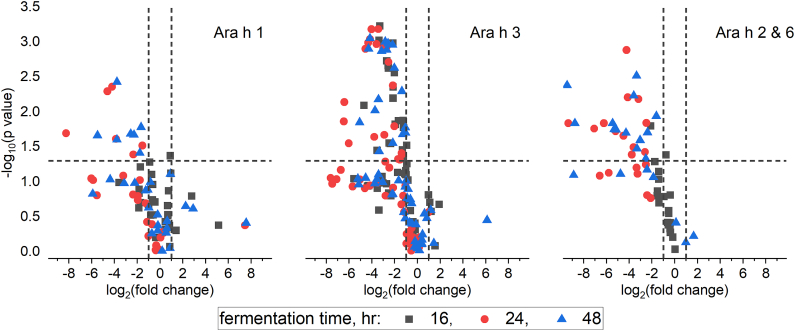
Log_2_-fold change plots of fermented to unfermented allergen peptide ratios in soluble protein extracts. Data were analyzed using label-free quantitation (replicate protocol) in Mascot Distiller. Data points with 2-fold or greater ratio changes lie outside the two vertical lines (−1 and 1), and data points falling above the horizontal line show statistical significance (p < 0.05).

Shotgun-MS analysis of protein extracts from 0, 16, 24, and 48 h of fermentation was done to further evaluate allergen changes during fermentation. Significant identifications of the major peanut allergens were obtained from search results of the soluble digests (supplementary file 2). Utilizing Mascot Distiller's identification-led quantitation, average (n = 4) relative intensities of peanut allergen peptides were calculated from the extracted ion chromatograms (0, 16, 24, and 48 h). Intensity ratios of peptides in fermented samples versus unfermented samples (0 h) were generated. The ratio values (expressed as binary logarithms), along with p-values obtained from two-sample t-tests, were used to construct volcano plots, shown in Fig. 3. Plots are shown for the three albumin families, with Ara h 2 and Ara h 6 peptides combined in one graph. At 16 h of fermentation, Ara h 3 displays significant (p ≤ 0.05) decreases in peptide intensities, while both Ara h 1 and Ara h 2/6 lack significant decreases. All four allergens show significant decreases in peptide intensities at 24 and 48 h of fermentation.

### 3.4 Secreted R. oryzae proteins observed by mass spectrometry

With increased fermentation time, proteins produced as a result of *R. oryzae* gene expression were observed at several size ranges. Gel slices (boxes 7–20) were searched against a *Rhizopus*-specific library; Table 2 summarizes the top identifications of *Rhizopus* proteins in the fermented samples. In most instances, the *Rhizopus* proteins were identified with less confidence than the peanut proteins, most likely due to the greater relative abundance of peanut proteins in the soluble fermented protein extract samples. Exceptions to this were seen at ∼125 kDa and 50 kDa, where pyruvate carboxylase (ADG65259.1) and an aldehyde dehydrogenase domain-containing protein (EIE85473.1) were identified with an extremely low probability that they were due to chance, respectively.

**Table 2:** Mascot scores and percent coverage values of top-ranking *Rhizopus* proteins identified in SDS-PAGE gel bands by LC-MS/MS analysis of tryptic digests (corresponding gel labels are shown in Fig. 2B). Percent coverage (% cov) indicates the percent of amino acids within the full-length protein that were assigned by high confidence spectral data.

32 h samples	protein *(accession)*		48 h samples	protein *(accession)*		
	score	% cov		score	% cov	
**7**	Pyruvate carboxylase *(ADG65259.1)*		**7**	hypothetical protein *(KAG0732957.1)*		
	518	17		24	4	
**8**	Aconitate hydratase, mitochondrial *(EIE81180.1)*		**8**	unidentified		
	88	9				
**9**	Heat shock protein 90-1 *(EIE86519.1)*		**9**	Putative Ssa4p *(CEJ00711.1)*		
	379	13		34	2	
**10**	Aldehyde dehydrogenase domain-containing protein *(EIE85473.1)*		**10**	Aldehyde dehydrogenase domain-containing protein *(EIE85473.1)*		
	1422	41		270	25	
**11**	Phosphoglycerate kinase 1 *(BAA01019.1)*		**11**	Phosphoglycerate kinase 1 *(BAA01019.1)*		
	365	31		91	11	
**12**	Glyceraldehyde-3-phosphate dehydrogenase *(EIE83328.1)*		**12**	Glyceraldehyde-3-phosphate dehydrogenase *(EIE81131.1)*		
	237	21		97	17	
**13**	Malate dehydrogenase *(ADG65261.1)*		**13**	Putative 40S ribosomal protein S0 *(CEJ00020.1)*		
	205	19		20	4	
**14**	Triosephosphate isomerase *(EIE78344.1)*		**14**	Triosephosphate isomerase *(EIE78344.1)*		
	110	17		58	10	
**15**	Peroxiredoxin-1 *(EIE77758.1)*		**15**	Thioredoxin peroxidase Tpx1 *(RCI01554.1)*		
	180	38		58	2	
**16**	Peptidyl-prolyl *cis*-trans isomerase A2 *(P0C1H8)*		**16**	Ricin B lectin domain-containing protein *(EIE86504.1)*		
	100	24		75	23	
**17**	C2 domain-containing protein *(PHZ09368.1)*		**17**	hypothetical protein *(KAG1051260.1)*		
	50	8		115	18	
**18**	Secreted protein *(EIE80346.1)*		**18**	Ribosomal protein L14e domain-containing protein *(RCI05826.1)*		
	89	13		26	4	
**19**	Glutaredoxin domain-containing protein *(EIE77184.1)*		**19**	unidentified		
	15	13				
**20**	unidentified		**20**	unidentified		
				

### 3.5 Immunoblot analysis with anti-peanut allergen antibodies

**Fig. 4:**
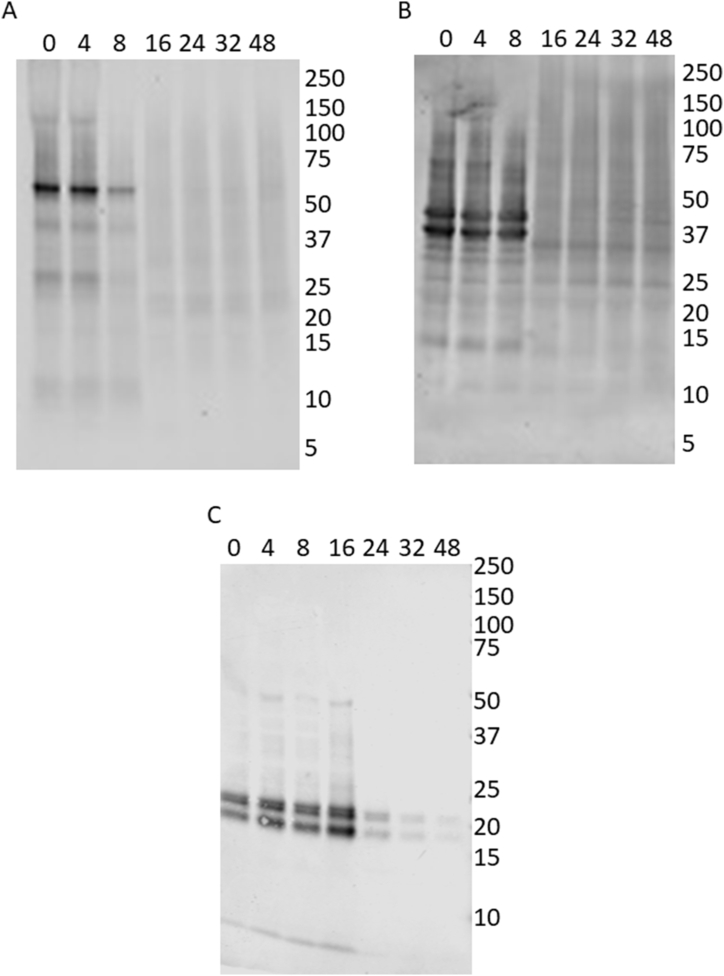
Immunoblots of *R. oryzae* fermented peanut protein probed with anti- Ara h 1 (A), anti-peanut (B), or anti-Ara h 2 (C) antibodies. Samples were fermented for the times (in hours) indicated at the top of each blot at 36 °C in a humidified chamber. Molecular mass markers (kDa) are indicated to the right of each blot.

Immunoblot analysis of peanut flour protein after *R. oryzae* fermentation also suggested a reduction in peanut allergens as fermentation time elapsed. Recognition of Ara h 1 by an anti-Ara h 1 antibody was reduced in intensity after 8 h of incubation and was missing by 16 h and at later time points (Fig. 4A). Similarly, recognition of the acidic subunit of Ara h 3 (bands migrating near the 37 kDa marker) and other peanut allergens by a polyclonal anti-peanut antibody were also reduced in intensity at the 16-h time point and beyond (Fig. 4B). Immunoblot analysis with a monoclonal anti-Ara h 2 antibody (doublet bands migrating just above and below the 20 kDa marker) indicated that it persisted until 16 h of fermentation, with a clear reduction in signal at 24 h and thereafter (Fig. 4C).

### 3.6 Anti-peanut allergen ELISA

**Fig. 5:**
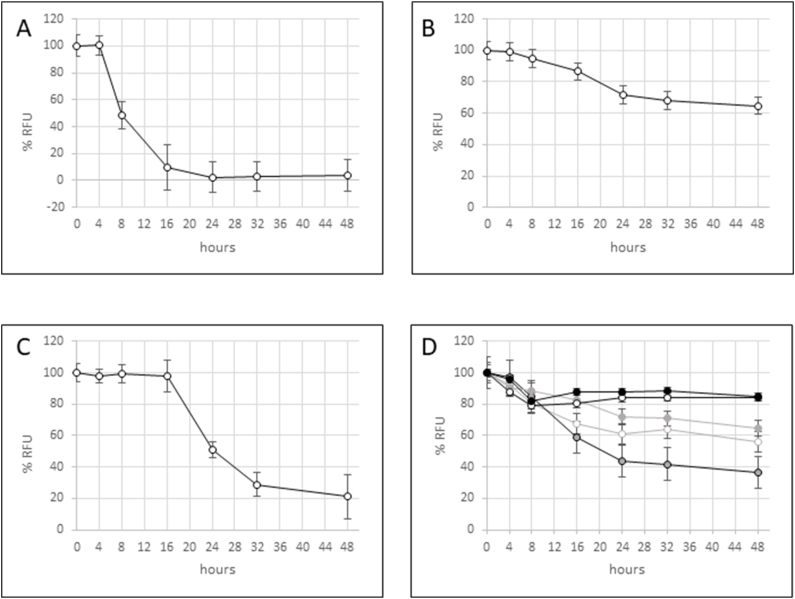
ELISA of *R. oryzae* fermented peanut protein probed with either anti- Ara h 1 antibody (A), anti-peanut antibody (B), anti-Ara h 2 antibody (C), or IgE from five peanut allergic volunteer samples (D). The percentage of relative fluorescence units (RFU) is indicated on the y-axis while fermentation time is indicated on the x-axis. The average of at least four biological replicates were plotted with standard deviation indicated by error bars.

Peanut flour fermentation samples were also evaluated by ELISA with anti-peanut antibodies and peanut allergic volunteer IgE. Signal from a direct ELISA using the anti-Ara h 1 antibody (Fig. 5A) confirmed the rapid reduction of Ara h 1 binding observed in the immunoblot after 8 h of fermentation, and recognition of Ara h 1 near the detection limit was observed after 16 h and beyond. Similarly, ELISA signal using the anti-Ara h 2 monoclonal antibody gradually decreased to approximately 20 % of the initial signal, with a slower onset compared to Ara h 1 binding dynamics (Fig. 5C). While a decrease in ELISA binding with the anti-peanut polyclonal antibody was also observed, binding was only reduced approximately 40 % compared to the initial signal (Fig. 5B). Fig. 5D shows ELISA results using volunteer anti-peanut allergic plasma. The reduction in IgE binding due to reduced allergen recognition is not uniform across volunteers and is only relatively slightly reduced by fermentation.

## 4 Discussion

A recent meta-analysis on the effects of various food-processing methods suggests that fermentation is a useful method to reduce allergen content in foods containing peanuts, tree nuts, and legumes [25]. Fermentation of soybeans has been used for decades to generate many different products including miso, natto, soy sauce, tofu, tempeh, and others. Studies of soybean fermentation with *Lactobacillus plantarum*, *Bacillus subtilis*, *Aspergillus oryzae*, or *R*. *oryzae* have reduced IgE binding to varying extents depending on pre-treatment steps and the microorganism used for fermentation [18,19,26]. Fermented soybean meal resulted in lower IgE binding capacity, milder intestinal damage, and higher interferon-gamma levels compared to standard soybean meal in a murine model [27]. Further, administration of *Lactobacillus* species into a soybean sensitized mouse model resulted in the proliferation of dendritic cells, a more optimal balance of T-helper cell differentiation, and an improved gut microbiota balance compared to soybean allergic mice [28].

The observations presented here indicate that *R. oryzae* may efficiently metabolize peanut allergens during fermentation and can destroy some antibody epitopes. However, the resulting protein fragments and peptides retain considerable peanut allergic IgE antibody binding. As shown in the mass spectrometric analyses of gel digests, significant Ara h 1 and Ara h 3 peptides were identified in samples fermented for 48 h with high confidence. Changes in peptide intensity ratios (Fig. 3) suggest an overall decrease in intact allergen proteins relative to unfermented samples. It should be acknowledged that this analysis did not account for any novel, non-tryptic peptides generated by fermentation. Further, fungal fermentation using a different fungi or with different conditions and/or time durations could produce unique results that may more comprehensively alter peanut allergen content.

Decreases in intact Ara h 1, Ara h 3, and Ara h 2 during *R. oryzae* fermentation were evident from SDS-page and immunoblot analyses (Fig. 2, Fig. 3). Qualitative decreases were also seen for signal representing cumulative recognition of each of the immunodominant peanut allergens (Fig. 5). Comparing Fig. 5C with 4A and 4B, Ara h 2 signal is slower to decrease relative to Ara h 1 and Ara h 3. This difference in relative reduction rates during fermentation is also seen in the mass spectral results (Fig. 4) and may suggest a greater resistance of Ara h 2 to fungal catabolism compared to Ara h 1 and Ara h 3.

While ELISA results with the anti-Ara h 1 monoclonal antibody (Fig. 5A) correlate well with immunoblot results, ELISA signal using an anti-peanut polyclonal antibody (Fig. 5B) is reduced by less than 40 % after 48 h of fermentation. ELISA results with anti-Ara h 2 monoclonal antibodies (Fig. 5C) display reduced signal with increased fermentation time, but a non-negligible amount of protein binding is still observed after 48 h of fermentation. While the immunoblot and ELISA data may seem contradictory in evaluations of anti-peanut polyclonal antibody binding, these differences in signal changes may be the result of different protein transfer efficiencies between gels and blots, which does not factor into ELISA measurements. The human anti-peanut IgE antibodies show a wide range of reactivity with the fermented peanuts but lack a reproducible decrease in IgE binding that would be necessary to consider fermented peanut products safe for consumption by peanut-allergic individuals. Studies of peanut fermentation using *Bacillus subtilis* and *Bacillus natto* have demonstrated efficient catabolism of peanut allergens and variable reductions in peanut allergic IgE binding [29]. Similar results were observed following fermentation of raw crushed peanuts with both *Bacillus natto* and *Lactobacillus plantarum*, however pretreatment by autoclaving enhanced the reduction in peanut allergic IgE binding [30]. ELISA results from Pi et al. (2022) indicated a 90 % decrease in IgE binding to autoclaved peanuts fermented with both *Bacillus natto* and *Lactobacillus plantarum* [30]. SDS-PAGE analyses in these studies demonstrate a clear reduction in the Ara h 1 and Ara h 3 proteins, but also reveal that at least some of the Ara h 2 and Ara h 6 (another 2S albumin) allergens survived the autoclave and fermentation treatments [30].

There are parallels between these studies with fermented peanuts and similar studies analyzing fermented tree nuts. For example, in an analysis of five dairy-free cheeses (brie, blue, cheddar, herb, and red) made with cashew nuts and quinoa, cashew allergen content was observed to decrease between 29 and 66 % using a commercial ELISA test kit after 72 h of fermentation with a variety of primarily lactic acid bacteria, depending on the type of cheese [31]. Most of the decrease in antibody binding was observed within the first 48 h, but individual cashew allergen content was not assessed [31]. Analogously, an evaluation of a commercially available lactic acid bacterial fermented cashew nut-based yogurt was shown to have reduced Ana o 1 and Ana o 2 (vicilin and legumin protein) allergen content [32]. However, similar to the fermented peanut studies described above, the cashew nut Ana o 3 allergen, a homolog of the peanut 2S albumin allergen Ara h 2, was not metabolized well by the lactic acid bacteria in the yogurt [32].

In contrast, our results indicate that the Ara h 2 (2S albumin) allergen was efficiently metabolized by *R. oryzae*. While the breakdown of Ara h 2 was slower than that of Ara h 1 and Ara h 3, immunoblot analysis indicated a clear change in antibody-Ara h 2 binding by 24 h of fermentation. Although fermentation observably hydrolyzed the Ara h 1, 2, and 3 peanut allergens to some extent, the IgE reactivity was not uniformly decreased when using five different volunteer anti-peanut allergic sera. This indicates that *R*. *oryzae* fermented peanut flour under these conditions is not suitable for individuals with peanut allergy.

Mass spectrometric analysis of tryptic peptides provides an opportunity to gain insights into the relative stability of the peanut allergens. The peptides shown in Fig. 4, which have x values less than 1 and y values greater than 1.3 (p ≤ 0.05), were significantly decreased in fermented peanut samples relative to unfermented peanut samples. Of the eight distinct peptides of Ara h 1 that fall into this category, four overlap with published linear IgE-binding epitopes [33]. In the case of Ara h 3, none of the significantly decreased peptides fall within published IgE-binding epitope regions. Two of the four published Ara h 3 epitopes [34] decrease as fermentation time progresses, but the decrease was not statistically significant. Ara h 2 and Ara h 6 are small, relatively compact allergens with eight and multiple linear IgE-binding epitopes [35,36], respectively. Both Ara h 2 and Ara h 6 have six significantly decreased peptides that overlap with the published epitopes (see supplementary file 3). The relative observations from the mass spectral data may help to explain the difference in signal reduction between immunoblots and ELISAs with anti-peanut polyclonal antibodies. While the reduction in Ara h 3 was clear based upon SDS-PAGE, the mass spectrometric data suggest that the IgE-binding epitopes of Ara h 3 were not significantly affected during fermentation. In contrast, Ara h 1 and Ara h 2 both show decreased levels of peptides corresponding to IgE-binding epitopes.

## 5 Conclusion

While the SDS-PAGE analysis indicated that *R. oryzae* may efficiently catabolize peanut allergens into smaller fragments during fermentation, the mass-spectrometry and ELISA results indicate that the resulting protein fragments and peptides retain considerable peanut allergic IgE antibody binding. All five peanut allergic sera retained IgE peanut allergen binding after 48 h. Further, peptides from Ara h 1, Ara h 2, and Ara h 3 were identified in the 48 h fermented samples with high confidence. While fermentation of nuts and plant-based proteins offers an alternative processing treatment to potentially enhance sensory properties or increase potential health benefits, the findings presented here indicate that products resulting from *R. oryzae* fermentation of peanut flour using these conditions should not be considered safe for individuals suffering from peanut and/or tree nut allergies. Perhaps future studies can use this information to guide the rational design of novel fermented peanut products that are hypoallergenic or could potentially be used as a therapeutic.

## Funding

This research was supported with funds from the USDA-ARS to C.P.M. (CRIS project 6054-43440-046-00D), and by an ARS Research Participation Program administered by the 10.13039/100006229Oak Ridge Institute for Science and Education (ORISE) through an interagency agreement between the U.S. Department of Energy (DOE) (DE-SC0014664) and the USDA. ORISE is managed by Oak Ridge Associated Universities (ORAU) under DOE contract number DE-SC0014664. All opinions expressed in this paper are the author's and do not necessarily reflect the policies and views of USDA, ARS, DOE, or ORAU/ORISE. Mention of trade names or commercial products in this publication is solely for the purpose of providing specific information and does not imply recommendation or endorsement by the USDA. The USDA is an equal opportunity provider and employer.

## Ethics declarations

Plasma samples for this study were collected at Plasma Lab International (Everett, WA, USA), a United States Food and Drug Administration (FDA) licensed facility (Establishment License Number 1050) after obtaining informed consent from each volunteer donor. Plasma samples were collected according to the US-FDA Code of Federal Regulations Title 21 by apheresis using an Aurora collection instrument with the inclusion of sodium citrate as an anticoagulant. This study was reviewed and approved by United States Food and Drug Administration (FDA) with the approval/license number: 1050 (dated Oct 2, 2023). All participants (or their proxies/legal guardians) provided written informed consent for the publication of their anonymized case details and images.

## Data availability statement

There is no research related data stored in publicly available repositories, and the data in this paper is available in the supplementary files and will be made available on request to the corresponding author.

## CRediT authorship contribution statement

**Christopher P. Mattison:** Writing – review & editing, Writing – original draft, Resources, Methodology, Funding acquisition, Formal analysis, Data curation, Conceptualization. **Rebecca A. Dupre:** Writing – review & editing, Writing – original draft, Methodology, Formal analysis. **Kristen Clermont:** Writing – review & editing, Investigation, Formal analysis. **John G. Gibbons:** Writing – review & editing, Validation. **Jae-Hyuk Yu:** Writing – review & editing, Validation.

## Declaration of competing interest

The authors declare that they have no known competing financial interests or personal relationships that could have appeared to influence the work reported in this paper.
