# Supplementary material for: Proteomic characterization of peanut flour fermented by Rhizopus oryzae
Source: Heliyon. 2024 Jul 21;10(15):e34793. doi: 10.1016/j.heliyon.2024.e34793 (PMC11320294; doi:10.1016/j.heliyon.2024.e34793)
Supplement: Multimedia component 3 [file mmc3.docx]

Supplementary file 3

Peanut allergen peptides derived from *R oryzae* fermentation overlapping with published IgE epitopes.

Peanut allergen sequences are shown below. Highlighted regions are IgE epitopes reported from linear peptide mapping studies for Ara h 1, Ara h 2, Ara h 3, and Ara h 6 [1-4], and bold amino acids indicate peptides had signal intensity values that were significantly decreased (the log2(intensity ratio) < -1 and t-test p-value < 0.05 (-log10(0.05) = 1.31)) in fermented samples relative to unfermented samples.

ABL14270.1 ara h 3

MAKLLELSFCFCFLVLGASSISFRQQPEENACQFQRLNAQRPDNRIESEGGYIETWNPNNQEFECAGVALSRLVLRRNALR**RPFYSNAPQEIFIQQGR**GYFGLIFPGCPSTYEEPAQQGRRYQSQRPPRRLQEEDQSQQQQDSHQKVHRFNEGDLIAVPTGVAFWLYNDHDTDVVAVSLTDTNNNDNQLDQFPR**RFNLAGNHEQEFLR**YQQQSRQSRRRSLPLSPYSPQPGQEDREFSPQGQHGRRERAGQEQENEGGNIFSGFTSEFLAQAFQVDDRQIVQNLRGENESEEQGAIVTVKGGLRILSPDRKSPDEEEEYDEDEYAEEERQQDRRRGRGSRGSGNGIEETICTATVKKNIGR**NRSPDIYNPQAGSLKTANELNLLILRWLGLSAEYGNLYRNALFVPHYNTNAHSIIYALR**GRAHVQVVDSNGNR**VYDEELQEGHVLVVPQNFAVAGK**SQSENFEYVAFK**TDSRPSIANLAGENSFIDNLPEEVVANSYGLPR**EQARQLKNNNPFKFFVPPFQQSPRAVA

AAA60336.1 ara h 1

MRGRVSPLMLLLGILVLASVSATQAKSPYRKTENPCAQRCLQSCQQEPDDLKQKACESRCTKLEYDPRCVYDTGATNQRHPPGERTRGRQPGDYDDDRRQPRREEGGRWGPAEPREREREEDWRQPREDWRRPSHQQPRKIRPEGREGEQEWGTPGSEVREETSR**NNPFYFPSR**RFSTRYGNQNGRIRVLQRFDQRSKQFQNLQNHR**IVQIEARPNTLVLPK**HADADNILVIQQGQATVTVANGNNRKSFNLDEGHALRIPSGFISYILNRHDNQNLRVAKISMPVNTPGQFEDFFPASSRDQSSYLQGFSR**NTLEAAFNAEFNEIRRVLLEENAGGEQEER**GQRRRSTRSSDNEGVIVKVSKEHVQELTKHAKSVSK**KGSEEEDITNPINLR**DGEPDLSNNFGRLFEVKPDKKNPQLQDLDMMLTCVEIK**EGALMLPHFNSK**AMVIVVVNKGTGNLELVAVRKEQQQRGRREQEWEEEEEDEEEEGSNREVRRYTARLKEGDVFIMPAAHPVAINASSELHLLGFGINAENNHR**IFLAGDKDNVIDQIEK**QAKDLAFPGSGEQVEKLIKNQRESHFVSARPQSQSPSSPEKEDQEEENQGGKGPLLSILKAFN

AAL37561.1 ara h 6

MAKSTILVALLALVLVAHASAMRRERGRQGDSSSCERQVDR**VNLKPCEQHIMQRIMGEQEQYDSYDIR**STRSSDQQQR**CCDELNEMENTQRCMCEALQQIMENQCDR**LQDR**QMVQQFK**R**ELMNLPQQCNFR**APQR**CDLDVSGGR**C

ABL14268.1 ara h 2

MAKLTILVALALFLLAAHASAR**QQWELQGDR**R**CQSQLERANLRPCEQHLMQK**IQRDEDSYGRDPYSPSQDPYSPSQDPDRRDPYSPSPYDRRGAGSSQHQER**CCNELNEFENNQRCMCEALQQIMENQSDR**LQGRQQEQQFKRELRNLPQQCGLRAPQR**CDLEVESGGRDRY**

References

1. Burks, A.W., et al., *Mapping and mutational analysis of the IgE-binding epitopes on Ara h 1, a legume vicilin protein and a major allergen in peanut hypersensitivity.* European journal of biochemistry / FEBS, 1997. **245**(2): p. 334-9.

2. Rabjohn, P., et al., *Molecular cloning and epitope analysis of the peanut allergen Ara h 3.* The Journal of clinical investigation, 1999. **103**(4): p. 535-42.

3. Otsu, K., R. Guo, and S.C. Dreskin, *Epitope analysis of Ara h 2 and Ara h 6: characteristic patterns of IgE-binding fingerprints among individuals with similar clinical histories.* Clin Exp Allergy, 2015. **45**(2): p. 471-84.

4. Stanley, J.S., et al., *Identification and mutational analysis of the immunodominant IgE binding epitopes of the major peanut allergen Ara h 2.* Archives of biochemistry and biophysics, 1997. **342**(2): p. 244-53.
